# Communicating scientific uncertainty in a rapidly evolving situation: a framing analysis of Canadian coverage in early days of COVID-19

**DOI:** 10.1186/s12889-021-12246-x

**Published:** 2021-11-29

**Authors:** Gabriela Capurro, Cynthia G. Jardine, Jordan Tustin, Michelle Driedger

**Affiliations:** 1 Department of Community Health Sciences, University of Manitoba, S113-750 Bannatyne Ave., Winnipeg, MB R2M 3Y9 Canada; 2 Faculty of Health Sciences, University of the Fraser Valley, 45190 Caen Ave., Chilliwack, BC V2R 0N3 Canada; 3 School of Occupational and Public Health, Ryerson University, 350 Victoria St, Toronto, ON M5B 2K3 Canada

**Keywords:** Infectious disease, News media, Public health, Risk communication; pandemic

## Abstract

**Background:**

The COVID-19 pandemic brought the production of scientific knowledge onto the public agenda in real-time. News media and commentators analysed the successes and failures of the pandemic response in real-time, bringing the process of scientific inquiry, which is also fraught with uncertainty, onto the public agenda. We examine how Canadian newspapers framed scientific uncertainty in their initial coverage of the COVID-19 pandemic and how journalists made sense of the scientific process.

**Methods:**

We conducted a framing analysis of 1143 news stories and opinion during the first two waves of the COVID-19 pandemic. Using a qualitative analysis software, our analysis focused, first, on how scientific uncertainty was framed in hard news and opinion discourse (editorial, op-ed). Second, we compared how specialist health and science reporters discussed scientific evidence versus non-specialist reporters in hard news and columns.

**Results:**

Uncertainty emerged as a “master frame” across the sample, and four additional framing strategies were used by reporters and commentators when covering the pandemic: (1), evidence -focusing on presence or absence of it-; (2) transparency and leadership -focusing on the pandemic response-; (3) duelling experts – highlighting disagreement among experts or criticizing public health decisions for not adhering to expert recommendations-; and (4) mixed messaging -criticizing public health communication efforts. While specialist journalists understood that scientific knowledge evolves and the process is fraught with uncertainty, non-specialist reporters and commentators expressed frustration over changing public health guidelines, leading to the politicization of the pandemic response and condemnation of elected officials’ decisions.

**Conclusions:**

Managing scientific uncertainty in evolving science-policy situations requires timely and clear communication. Public health officials and political leaders need to provide clear and consistent messages and access to data regarding infection prevention guidelines. Public health officials should quickly engage in communication course corrections if original messages are missing the intended mark, and clearly explain the shift. Finally, public health communicators should be aware of and more responsive to a variety of media reporters, who will bring different interpretative frames to their reporting. More care and effort are needed in these communication engagements to minimize inconsistencies, uncertainty, and politicization.

## Introduction

In late January 2020, Canada identified its first case of COVID-19, a respiratory disease that was quickly becoming a Public Health Emergency of International Concern (PHEIC) [1]. The disease, caused by the novel coronavirus SARS-CoV-2, prompted a rapid public health response that included forced lockdowns for several weeks across Canada. As elected officials and public health authorities struggled to contain the spread, scientists worldwide raced to understand the transmission and symptoms of the illness and develop a vaccine.

With no prior knowledge nor immunity against the novel coronavirus, public health authorities grappled with uncertainty, i.e. uncertainty about the validity of claims based on scientific evidence or claims communicated by scientists [2]. As new evidence emerged, policies and guidelines were frequently revised and communicated to the population, emphasizing that uncertainty. Scientific research, risk management policy decisions, and risk communication happened simultaneously. News media and commentators analysed the successes and failures of the pandemic response in real-time, bringing the process of scientific inquiry, which is also fraught with uncertainty, onto the public agenda.

We examine how Canadian newspapers communicated scientific uncertainty in their initial coverage of the COVID-19 pandemic and how journalists – both health reporters and those who do not usually cover health and science topics – made sense of the scientific process.

## Background: scientific knowledge, uncertainty and the precautionary principle

News media are a dominant source of health information for the general public and most people learn about novel health threats through reading the news [3]. News coverage of diseases is also associated with public perception of the severity and prevalence of the risk [4]. The COVID-19 pandemic has been a main focus of news coverage worldwide since the virus was first reported in December 2019, including the evolution of scientific knowledge about the virus.

Scientific knowledge is generally understood as neutral representations of reality discovered through observations of the natural world [5]. However, in reality, the scientific process is a complex, nonlinear endeavor [3]. Scientific knowledge is premised on the best available evidence, and it is continuously revised in light of new evidence [6]. Furthermore, what counts as legitimate medical knowledge and practice is inseparable from the sociocultural context in which they are produced and experienced [7]. For example, scientific knowledge about the origins of the SARS-CoV-2 virus, how it spreads, infection symptoms, and how to minimize infection risk have all evolved and been interpreted in different ways [8, 9].

Novel risks, such as COVID-19, generate feelings of uncertainty, and many competing narratives emerge that seek to define the risk, determine preventive measures, and identify vulnerable groups [10, 11]. These narratives are then reproduced by news media, which are important sources of science and health information for wider audiences [12]. News coverage also impacts public understanding of risks and affects the adoption of risk prevention behaviours [13–15]. Uncertainty, a key aspect of scientific knowledge production, is also highlighted in news coverage of risk. Additionally, scientists, politicians, reporters and other social actors, construct and evoke uncertainty in different ways [16] when advancing knowledge claims. Scientific uncertainty has also led public health experts [17] and health advocates [18] to argue in favour of invoking the precautionary principle in health policy-making. The precautionary principle states that “complete evidence of a potential risk is not required before action is taken to mitigate the effects of the potential risk” [19]. In the context of a global pandemic, fear may intensify public calls to adopt the precautionary principle, through which infection preventive measures are adopted even if there is insufficient evidence to support them.

Consequently, media coverage of scientific topics is often influenced by scientific research. Yet, it does not merely reproduce research findings. Rather, through the use of frames, it situates them in a context that makes the story resonate more with audiences [20, 21]. We conceptualize frames from a sociological perspective as the way in which people classify and interpret information, and construct meaning about everyday life [22]. Emphasis news frames, based in sociology, refer to how an issue is characterized in news reports, and how the information is organized and presented, which can influence public understanding of the issue [23]. Framing analyses have been conducted to understand, for example, how media messages affect public understanding of climate change, biotechnology, and some diseases (c.f [24–27].). In that sense, news media play a significant role in shaping and moderating how scientific uncertainty is communicated and how controversial issues are interpreted [2, 28]. We also draw on the concept of “master frame” to refer to specific frames that are flexible enough to be articulated in a variety of contexts [29]. The concept of “master frame” was first used in social movement theory [25], and it is a useful analytical category for our study given that scientific uncertainty emerges as an ever-present frame in the news coverage of COVID-19. In news discourse, scientific uncertainty is also used by different actors to justify specific actions and to discredit others [16].

The COVID-19 pandemic has generated much uncertainty and news media have brought forward issues typically discussed within academic circles, such as scientific uncertainty, pre-print studies and the value of peer-review, the validity of data and clinical trials, and so forth. Recent studies have criticized news media and policy-makers for not communicating openly about scientific uncertainty about COVID-19 [30, 31], arguing that lack of transparency about uncertainty can erode trust [8, 32]. We examine how reporters grappled with the intrinsic scientific uncertainty of the novel risk, specifically how they framed uncertainty, to further our understanding of journalistic practice during health crises and dominant framing strategies. Based on this analysis we also advance risk communication recommendations to manage the interpretative breadth of these frames.

## Method

We conducted a framing analysis [23, 33] of Canadian newspaper print and online coverage of COVID-19 to examine, first, how newspapers communicated scientific uncertainty (RQ1); and, second, how journalists – both health reporters and those who do not usually cover health and science – made sense of the scientific process (RQ2). To address our first question, we examined how scientific uncertainty was framed in hard news and opinion discourse (editorial, op-ed). For our second research question, we compared how specialist health and science reporters discussed scientific evidence versus non-specialist reporters in hard news and columns. We determined whether a journalist was a specialist science or health reporter by reviewing their profile in their newspaper’s website, as well as their personal website. Reporters were considered specialists if they were described as the science or health reporter for their daily, or if they had previously done health and science reporting, which would give them expertise on the topic. We define non-specialist reporters as carrying their expertise and understanding for the types of stories they dominantly write and who may grapple with uncertainty in relation to their focus on politics, the economy, sports, lifestyle, and the like. However, non-specialist reporters may be less familiar with the iterative and provisional qualities foundational to scientific inquiry and what constitutes solid evidence. Consequently, this may be reflected in their reporting of COVID-19.

Two newspapers have national distribution, *The Globe and Mail* and the *National Post.* The other five newspapers have a more regional focus, *Montreal Gazette, The Toronto Star*, *Ottawa Citizen*, *Winnipeg Free Press*, and *Vancouver Sun*. These dailies were selected because they offer both national and regional perspectives, and they are owned by different corporations,^1^ which could result in different viewpoints being promoted in their coverage. We sampled for six different topics: (1) Travel, isolation and quarantine; (2) Epidemiological models; (3) Testing; (4) Use of face masks; (5) Physical distancing; and (6) Airborne transmission. These topics were selected due to their salience at specific times during the pandemic, expressed in volume of news stories and opinion pieces, and because they involved shifts in public health guidelines caused by scientific uncertainty.

The sample was gathered from the database Factiva using various combinations of the keywords “COVID-19” “Covid19” “coronavirus” “quarantine” “isolation” “travel” “model*” “guideline” “test*” “mask*” “aerosol*” “airborne” “distancing”. Our sample includes news coverage between March and mid-July, 2020. However, given that each topic became relevant in different moments, we collected articles for each of them using the most relevant date range. We defined relevant date ranges to include roughly one week before and following a specific date where there was a tipping point in guidance communication to monitor shifts in the uses of scientific uncertainty and evidence. Each topic’s sample encompasses at least two weeks of coverage to a maximum of 10 weeks (see Table 1). News articles that mentioned these topics only tangentially were not considered for analysis. The sample was composed of 1143 articles (see Table 2).

**Table 1:** Dataset sampling frame by topic, date, keywords, and outputs

Topic	Dates covered	Keywords	Number of articles
Travel Quarantine Isolation	March 5th- March 31st	[“Travel”] and [“quarantine” or “isolation”] and [“Covid-19” or “Covid19” or “coronavirus”]	222 (19%)
Epidemiological models	April 1st- April 30th	[“Model*” or “modelling” or “projection*”] and [“Covid-19” or “Covid19” or “coronavirus”]	148 (13%)
Testing	May 5th- May 25th	[“Test*”] and [“Covid-19” or “Covid19” or “coronavirus”]	106 (9%)
Face masks	March 1 - July 14th	[“Mask*” or “face mask”] and [“Covid-19” or “Covid19” or “coronavirus”]	374 (33%)
Physical distancing	March 20th – April 4th	[“Distancing” or “social distance” or “physical distance”] and [“Covid-19” or “Covid19” or “coronavirus”]	255 (22%)
Airborne transmission	May 1st – July 15th	[“indoor transmission” or “aerosol*” or “airborne” or “microdroplet*”] and [“Covid-19” or “Covid19” or “coronavirus”]	38 (3%)

**Table 2:** Distribution of news stories written by specialist and non-specialist reporters. Topics: Travel, quarantine and isolation (TQI); EM (Epidemiological models); Facemasks (FM); Testing (TT); Social distancing (SD); Airborne Transmission (AT)

Topic	Specialist	Non-specialist	Columnist	Editorial	medical expert	Wire	N/A	Total
TQI	7	165	15	6	2	26	1	222
EM	19	102	15	3	3	5	1	148
FM	31	218	44	6	13	62	0	374
TT	10	69	3	1	4	19	0	106
SD	16	177	30	5	8	17	2	255
AT	5	24	3	0	2	4	0	38
Total	88	755	110	21	32	133	4	1143

We uploaded the reports to the qualitative analysis software NVivo12. After an initial reading of the sample, we developed a codebook with five principal codes: uncertainty; evidence; shifting guidelines; disagreement among experts; and trust. These codes corresponded to the most recurrent themes in the sample. The coding process was open, allowing for new codes to emerge during the analysis. We also coded the news stories for type of story (hard news, opinion, letter to the editor, or other), geographical focus (local, regional, national, international), and whether the reporter was classified as a specialist science/health journalist, a columnist, or an expert writing an op-ed. Coding was done using NVivo12, which allows for an open coding process and facilitates the organization and cross reference of data. While we did not use the software’s automated coding features, we did use of the software’s functionality to perform searches of our coded data, for example to find all the coded excerpts where two or more codes intersected. The analysis was purely researcher driven, the decisions as to what constituted a “specialist reporter” or topic (“use of facemasks”) was done manually by the lead author using the software to organize, manage and retrieve content.

We coded one topic at the time to ensure that the coding process was focused on how scientific evidence was discussed in each specific case, although we acknowledge that there was often overlap between topics in a particular story. We resolved disagreements about coding and the classification of reporters as specialists or not through discussion. For the sample subset (*n* = 31) where double coding was completed, the final overall Cohen’s kappa coefficient for intercoder reliability was .97. Once the coding process was complete, we examined the data in each code and followed an inductive process to identify the most salient interpretive frames used by reporters.

## Results

Scientific evidence and uncertainty were central points in the news coverage of the COVID-19 pandemic in Canada. Overall, there was consensus expressed in hard news and opinion pieces that the public health response to COVID-19 needed to be evidence-based. These same reporters and commentators also lamented that public health authorities and elected officials did not apply the precautionary principle. This ambivalence regarding the uses and application of scientific evidence and uncertainty emerged in the news coverage as a “master frame.” Within this master frame, reporters and commentators used four framing strategies (see Table 3) focusing on (1) evidence; (2) transparency and leadership; (3) duelling experts; and (4) mixed messaging, which they employed to support or challenge decision-making in the name of scientific uncertainty. Below we present these results and address differences in how health and science reporters framed scientific uncertainty compared to non-specialist reporters.

**Table 3:** Frame descriptions

FRAME	DESCRIPTION
Uncertainty	Master frame across the sample. From uncertainty about the virus to uncertainty about the appropriateness of the pandemic response.
Evidence	Demands for the pandemic response to be evidence-based, despite acknowledging the uncertainty that surrounds a novel threat.
Transparency and leadership	Policy decisions and evolving public health response to the pandemic became politicized and interpreted as successes and failures in leadership and transparency.
Duelling experts	Conflicting expert opinions to highlight uncertainty or use of expert opinions to criticize public health decisions perceived as not being evidence-based.
Mixed messages	Frustration expressed over evolving and constantly changing guidelines, leading to criticism for confusing public health communication.

### Evidence

COVID-19 has different aetiological and epidemiological characteristics than other known coronaviruses (e.g., SARS, MERS). Given its novelty, many reporters and commentators, particularly in newspapers with national distribution, wrote about the need for an evidence-based response to the pandemic while also addressing the contingent nature of scientific knowledge and the importance of using the precautionary principle. Reporters followed new study results closely, both peer-reviewed and pre-prints, and cited experts and public health officials, who evaluated the emerging evidence’s significance. For example, a *Toronto Star* reporter cited an expert explaining the contingent nature of scientific evidence:

"We don't want people to think we are ignoring these issues. We're very aware of it," he told the Star. "If we see something compellingly different, we will act on it ... Science evolves. Knowledge evolves. And sometimes, the guidance, policies, implementation often take longer to evolve than sometimes the evidence does” [34]

These reporters brought to public attention debates about “the scientific method” and “validity” that are more customarily restricted to academic circles. For example, in several news stories and opinion pieces, it was explained that pre-print studies do not have the same validity as peer-reviewed ones. For example, a reporter with the *Toronto Star* referred to the system of peer-review in scientific research:

The paper was posted on a pre-print server, which meant it was not peer-reviewed, the gold standard by which the science community verifies studies by having other scientists look over the research. In a normal, pre-epidemic course of events, that paper would likely not have been released to the general public without having gone through such a review [35].

Some non-specialist journalists, who were also following new studies, occasionally reported pre-print studies as accepted scientific knowledge. For example, a fitness reporter for the *Globe and Mail* cautioned runners and cyclists about the possibility of spreading aerosolized droplets while exercising, based on the findings of a heavily criticized Belgian study that had not yet been peer-reviewed:

[R]esearchers concluded that runners and cyclists should maintain a distance of at least 10 metres when moving in a straight line because anyone behind them could be exposed to their fluids within that distance. It is important to note that the study (…) has not been peer-reviewed and has been heavily criticized since its release [36].

Some journalists and commentators explained that “anecdote is not evidence” and is therefore not enough to support changes in medical treatment or public health guidelines. “We should always be leery of people who claim to have miracle cures, especially when their “evidence” is published in the tabloid Daily Mail and not a reputable scientific journal” [37]. Additionally, health and science reporters, as well as experts writing opinion pieces, sought to explain the process of scientific knowledge production as nonlinear, and sometimes convoluted. For example, Professor Timothy Caulfield, expert in health and science communication, wrote in the *Globe and Mail:*

Remember science is a difficult and (usually) slow process. It is not a list of immutable facts. U-turns, retractions, nasty disagreements between experts and conflicting data are all frustrating, but, alas, that’s how science works. (…) During a pandemic, public health decisions often need to be made using a less-than-ideal body of evidence. And recommendations that are based on emerging science will (and should) evolve [38]..

Reporters also emphasised that updating public health guidelines is not exclusive to Canada, and other countries and health organizations do the same. This was in response to increasing public criticism as guidelines regarding use of facemasks and physical distancing evolved, as illustrated by health columnist, Andre Picard:

The Public Health Agency of Canada is not alone in adapting. The U.S. Centers for Disease Control and Prevention has been much more clear in its messaging, urging Americans to wear home-made masks. Even the World Health Organization, which has long been unenthusiastic about masks, has shifted its views [39].

Despite acknowledging the need for evidence-based policies and guidelines, non-specialized reporters and some experts often lamented that elected officials and public health officers were hiding behind scientific research. In those cases, arguments were made for why action, governed by a precautionary principle, was needed in the absence of concrete evidence. For example, a *Toronto Star* reporter referred to the SARS Commission report, conducted following the 2003 outbreak, explaining one of its main conclusions: “‘The point is not science, but safety,’ one chapter of the report reads. ‘We should be driven by the precautionary principle that reasonable steps to reduce risk should not await scientific certainty’” [40]. Others pointed to emerging scientific evidence of the efficacy of facemasks in slowing down the transmission of COVID-19 and criticized public health authorities for not using it to mandate the use of face masks in Canada. The Chief Public Health Officer of Canada, Dr. Theresa Tam, was criticized for not acting immediately on emerging evidence, thus framing the evolution of scientific knowledge as a linear process and research findings as absolute:

Research published in March suggested that undocumented infections (meaning not-yet-diagnosed, mildly symptomatic or asymptomatic carriers) were the source of nearly 80 per cent of documented COVID-19 cases. All of that material was published before Dr. Tam said, in late March, that ‘there is no need to use a mask for well people’ [41].

Dr. Tam faced much criticism for relying on the World Health Organization’s (WHO) guidelines and not recommending the closure of borders. An editorial in the *Globe and Mail* considered this a grave omission of emerging research, which was framed as a political problem. The editorial suggested that Canada’s Prime Minister should not have followed Dr. Tam’s recommendation, and that scientific evidence cannot be prioritized when so much uncertainty remains about the virus. Furthermore, the editorial refers to Dr. Tam as “Ms. Tam,” stripping her of her medical and public health expertise:

If this fast-moving pandemic has taught us anything, it is that elected officials need to respect the primacy of scientific evidence over politics. But there can be limits to that. The undeniable truth is that there is much about this coronavirus that is unknown (…) But imagine if Mr. Trudeau has said, in late January, that out of an abundance of caution he was going to limit flights between China and Canada, and impose an enforced two-week quarantine on anyone arriving in the country from the most affected areas, in spite of recommendations to the contrary from Ms. Tam [42].

The production and publication of epidemiological models also generated much media attention. Reporters cited experts who explained the usefulness of these models causing controversy over whether these models should be made public. In this discussion, models were framed in two very different ways: purveyors of hope vs. being as (un) reliable as fortune-telling. Models could quantify how population-level adherence to restricted measures contained the spread of COVID-19, offering hope of a return to ‘normal’ life, as explained in this *Toronto Star* story:

Ontario's top public health experts not only predicted how many people may die by month's end. More importantly, they also estimated how many have been saved to date and will be spared in the days to come (…) If society can maintain vigilance and social distancing, the latest modelling suggests we would be saving — sparing — 4,400 [lives] In other words, and in precise numbers, we are on track to reduce the death toll by 73 per cent [43].

Models were also described as being as unreliable as horoscopes because epidemiological models do not provide actual evidence but rather express probabilities based on the best (and often limited) evidence available at the time. A reporter for the *National Post,* for example, explained in a news story that simply feeding data to models does not guarantee accurate predictions if the data is not well managed and credible [44]. Models are equally described as paradoxical, confusing and contradictory.

### Transparency and leadership

There is a general acknowledgment in the analysed articles that the scientific understanding of this virus is continually changing as new aspects are learned; consequently, public health response measures are expected to be fluid. However, instead of framing these shifts as a normal part of scientific research and public health policy, non-specialist reporters framed these changes as political complacency and public health incompetence, thereby politicizing science. Two instances of this were the public debate over the use of face masks and whether federal and provincial governments should make epidemiological models public.

In the first weeks of the pandemic, Dr. Tam refused to recommend the general public wear facemasks because there was not enough scientific evidence to do so, while many experts and commentators argued the opposite. Weeks later, Dr. Tam finally conceded that wearing a facemask could offer an extra layer of protection and was heavily criticized in opinion pieces, first for rejecting the masks and then for changing her position. A *Toronto Star* reporter, for example, explained that “the new advice represents a change in Tam’s stance (…) and reflects what she said is rapidly changing scientific evidence about the transmission of the virus,” but also qualified the change as slow and Dr. Tam as defensive: “She defended the agency’s slowness to change its advice” [34].

Provincial public health officers also faced criticism for revising their initial message against the general use of facemasks. Reporters cited opposition leaders and medical and non-medical experts who accused provincial officers of being inconsistent. For example, a *Montreal Gazette* reporter who usually covers art and entertainment news explained that “McGill [University] law professor Daniel Weinstock also thinks the Quebec government has a share of the blame in Quebecers’ slow adoption of masks” and quoted the law expert saying

“At the beginning of the pandemic, [Quebec’s public health officer] Dr. Arruda was quite adamant, saying there was no need to wear a mask if you didn't have symptoms (…) For weeks and weeks, it was repeated. It's hard to undo that messaging overnight” [45].

Once there was general acceptance of the evidence in favour of wearing facemasks, and public health officers were recommending the general population to use them, the controversy shifted its focus to whether facemasks should be regulated. As local authorities mandated using masks, authorities in Quebec and Ontario were criticized for not making them mandatory at the provincial level. In the *Globe and Mail,* reporters usually writing news stories for the politics and international sections framed the lack of a province-wide mandate in Ontario as a failure of the provincial government:

Councillor Joe Cressy, who chairs Toronto’s board of health, said the province should be mandating masks with its emergency powers to avoid the ambiguity of a “patchwork” of bylaws and health orders. “In the absence of provincial action, the City of Toronto and other GTA municipalities will not delay,” Mr. Cressy said. “Doing nothing is no longer an option” [46].

Federal and provincial governments were often condemned for following the advice of public health officers and the WHO. In these pieces, reporters framed the WHO’s revised guidelines as attempts to correct mistakes, instead of revisions based on new evidence. One such case was when federal authorities lowered the level of PPE requirement for healthcare workers from N95 respirators to surgical masks, as shown in this *Toronto Star* story:

"It is preposterous to claim that surgical masks are sufficient protection," says Possamai. "What you have is a scientific approach in the public health agencies of Canada and Ontario that is really rigid and not open to new findings. They say the science is settled ... (But) they're ignoring a huge body of science that is growing and is very persuasive" [34].

Epidemiological modelling also became politicized as reporters demanded provincial authorities publish their models and framed it as an issue of transparency. The arguments by public health officers that sharing that data without proper contextualization was lost rapidly as the days went by and the frame of failed leadership became more prominent. When Ontario Premier Doug Ford decided to share the data, it was welcomed and became an expectation for other provincial and federal leaders to do the same. Transparency by one was conjecture by another (as illustrated in the second quote below). However, instead of focusing on whether there was enough data to provide a complete picture or whether it was in the public interest to share them, many political reporters saw the unwillingness to share the data as a lack of transparency and bad leadership.

Ontario is willing to show you what the federal government is not. After refusing to release projections for how the COVID-19 pandemic could play out in the coming weeks, Premier Doug Ford is promising full disclosure while Prime Minister Justin Trudeau says Canada won't release any potential scenarios until it's clearer which path the virus is likely to take [47].

[Manitoba] Premier Brian Pallister dismissed Ontario releasing its models and projections in the name of transparency. He pointed to an early model released by Ontario on April 6 that projected anywhere from 3,000 to 15,000 deaths in that province. “One could argue that's transparency. I would argue that's conjecture," said Pallister [48].

Many commentators and reporters demanded the immediate publication of the epidemiological data arguing that citizens have the right to know whether the imposed preventive measures, such as staying at home and keeping physical distance from others, were working and for how long the measures would remain in place. In the *National Post*, for example, economy columnist Terence Corcoran framed the publication of models as the government finally being able to provide evidence that the regulations, which had crippling repercussions on the national economy, were reducing the spread of COVID-19:

A full and frank exposition of the facts should not be expected from governments that have already adopted massive and unprecedented interventions into the economic and daily lives of every individual. The need to justify actions taken to date is likely to take precedent over a balanced review of the science and the options [49].

Other reporters and columnists lamented that the government did not collect more data to develop more precise models. A *Globe and Mail* editorial, for example, argued that “Canada has long-standing problems with data” and that “decisions are only as good as the information underpinning them” [50]. Chantal Hébert, political columnist for the *Toronto Star*, used a political metaphor to explain epidemiological modelling, thus evoking the more familiar scenario of federal elections, to clarify that epidemiological models are based on probability:

Think of the projections coming to light - in B.C., Ontario and soon in a host of other provinces - as you would the provincial breakdown of poll numbers in a federal election. (…) The same is as true when it comes to beating back the pandemic (…) The projections the provinces and Ottawa operate under are no more cast in stone than mid-campaign election polling numbers [51].

### Duelling experts

Since the beginning of the pandemic evolving scientific evidence and shifting guidelines caused frustration, and occasionally reporters focused on contradictory evidence. The ‘duelling experts’ frame has been commonly used in news media coverage of scientific issues that are socially controversial, such as climate change, by pitting two experts who disagree [52]. In our analysis we found that reporters used conflicting expert opinions to highlight disagreement among experts or to criticize public health decisions for not adhering to expert recommendations, in both cases emphasizing uncertainty.

One example of this was testing. Early in the pandemic, Canada initiated various lockdowns in the spring of 2020 to curtail spread in the first wave of COVID-19. With plans to reopen the economy across the country, experts in both Canada and the United States stressed the importance of creating a solid strategy for large-scale testing and contact tracing for any identified cases. Various provinces followed this advice over the summer, but as the cases continued to rise, the opposite occurred – public health measures were relaxed to alleviate the economic impact of the pandemic, and contact tracing efforts in some areas were entirely abandoned. There was heavy criticism in the news coverage of lack of testing capacity and whether the provinces were testing enough, particularly Ontario and Quebec, given expert warnings that without massive testing, it would be impossible to control the spread of COVID-19. Not enough testing was interpreted in the national and regional news coverage as incompetence, lack of transparency, bad management, and failing to meet basic public health guidelines. For example:

Epidemiological experts in Canada and the U.S. share two important common beliefs: The testing rates need to at least double before we reopen the economy broadly; and the only way to avoid a resurgence in cases is having the ability to test and trace swiftly, ideally within 24 hours, so further spread can be contained. We're not even remotely close to that standard in large swaths of the country [53].

Health columnist Andre Picard noted that “whatever the excuses, it’s clear we have not made testing and tracing a priority. We have not invested in the response that the urgency of the situation requires” [53]. In a different column, Picard went further in his criticism arguing that it was not only a matter of lack of resources but a lack of political accountability:

Sixteen weeks and almost 25,000 cases later, Ontario is still struggling to actually test people.[…]it’s unclear why testing targets are falling short. Is it lack of supplies such as swabs and reagents? Lack of laboratory capacity? Bureaucratic disorganization? The lack of transparency is appalling, the data gaps worse [54].

A *Montreal Gazette* health reporter also wrote about the lack of testing capacity in Quebec, stating that “some experts have criticized the program for failing to test asymptomatic individuals in the community, but it’s now evident that such random testing is a luxury authorities here can’t afford” [55]. These limitations in testing and contact tracing stopped the government of Quebec from reopening schools and retail stores.

Elected officials were also condemned for not following their own health experts’ advice. For example, politics columnist Robyn Urback referred to the decision to reopen Ontario’s economy early and against the recommendation of the Minister of Health:

In April, the province said it would need to see a ‘consistent two- to four-week decrease in the number of new daily COVID-19 cases’ to begin easing public-health measures. Instead, Ontario entered “Phase 1” of reopening in May after seeing barely a week of declining cases, for a reason Premier Doug Ford still has not articulated [56].

While criticism was met with silence by the government of Ontario, in British Columbia the Chief Provincial Health Officer, Dr. Bonnie Henry, wrote an op-ed in the *Vancouver Sun* explaining why the province was not widely testing the general population: “Many have asked and many continue to ask about who is getting tested for COVID-19 in B.C. and why we don’t just ‘test, test, test everyone.’ (…) we adapted our testing approach as we learned more about the virus and the test, and as more tests became available. We will continue to adapt as we progress through our pandemic response” [57].

Another topic that sparked public debate was that of airborne transmission. While COVID-19 is now known to be airborne [58], at the beginning of the pandemic it was believed to be transmitted mainly through droplets [59]. Some researchers, however, expressed concern at the beginning of the pandemic over the possibility of airborne transmission, i.e., transmission via microdroplets capable of travelling through the air a longer distance and time than initially thought. This topic rapidly became controversial as uncertainty remained over whether healthcare workers should be wearing N95 respirators. In an op-ed, two medical students and a professor ask whether the decision to downgrade the required PPE was science-based:

It’s still uncertain how health care workers can best protect themselves in a clinical setting during the pandemic. There have been no randomized trials, the most rigorous form of evidence, comparing surgical masks to N95 respirators for COVID-19 [60].

The controversy over airborne transmission involved mostly experts, while elected officials and the general public mainly remained on the margins. In hard news and opinion pieces, reporters and commentators explained why there was disagreement among experts and contradictory research findings. The controversy erupted after “in an open letter to the WHO, 239 scientists in 32 countries have outlined the evidence showing that smaller particles can infect people and are calling for the agency to revise its recommendations” [61]; to which the WHO responded that the evidence was unconvincing [53]. This disagreement brought again to the forefront the scientific process, the importance of peer-review, the need for replicability of studies, the difference between conducting an experiment in a laboratory versus the reality in the community, and so forth. For example, in an op-ed, Professor Caulfield explained that much of the scientific controversies around COVID-19 were an expected side-effect of the unprecedented amount of research:

While it is inspiring to see the research community respond so vigorously to the pandemic crisis, all this activity has also created a churning sea of bad data, conflicting results and hyped headlines. One day a study, published in a renowned biomedical journal, is being hailed as definitive data that should (and does) guide our actions and policies. The next day it is retracted (or being asked to be retracted). Even the experts are struggling to agree [38].

Given this scientific uncertainty and the disagreement among experts, which the *Globe and Mail* qualified as “raging” [62], many researchers and health experts were quoted in the news coverage asking the WHO to adopt a precautionary principle. In a news story, health reporter Carly Weeks quoted a physician explaining the principle that even if the evidence is not convincing, it does not mean that airborne transmission is not happening: “‘There is no incontrovertible proof that SARS-CoV-2 travels or is transmitted significantly by aerosols, but there is absolutely no evidence that it’s not,’ said Dr. Trish Greenhalgh” [54]. A few days after the experts published their letter regarding airborne transmission, the WHO acknowledged airborne transmission reports. The organization, however, still did not call the virus airborne [63].

This debate shined a spotlight on expert disagreement. Those who argued for calling COVID-19 airborne and adopting tighter infection prevention measures sought for a broader definition of “airborne” to include instances of aerosolization. The WHO, however, follows a narrower definition of “airborne” to describe illnesses such as measles, one of the most contagious infectious diseases. In the news coverage, the debate focused on the validity of the studies producing new evidence and the fact that new evidence makes scientific knowledge progress. At the same time, some experts were quoted explaining the semantic nature of the debate:

"To the general public, the word (airborne) can be pretty confusing because it suggests that COVID is gonna come through the keyhole and get you in your sleep. And well, it isn't," said Colin Furness, an epidemiologist with the University of Toronto. "No one is suggesting COVID behaves anything like measles … That's not the point (the scientists) are trying to make" [64].

Eventually, on November 4th, 2020, the Public Health Agency of Canada quietly updated its guidelines on how COVID-19 spreads, acknowledging the risk of transmission via aerosols [65].

### Mixed messaging

Another source of frustration expressed in the news coverage of COVID-19 was the frequent change in guidelines and confusing public health communication, an accusation launched against many provincial and federal public health authorities across Canada. For example, initially, there was much confusion and uncertainty regarding isolation guidelines for travellers, which later evolved into confusion about who should isolate, when, and how.

A science reporter with the *Toronto Star* expressed confusion about safe distancing guidelines. “The public has been firmly instructed, sometimes even scolded, to remain inside for all but essential outings to slow the spread of COVID-19. We know physical distancing saves lives,” the reporter acknowledged only to later ask: “So is going for a walk an ‘essential outing,’ or a reckless luxury?”; to which she replied: “Just use your common sense” [66]. A reporter with the *National Post* expressed a similar argument that “self-isolation doesn’t work if it’s left to each person to interpret loosely what the concept means; you can’t have successful disease control with exceptions and fuzzy half-measures” [67]. The *Globe and Mail* reported another discrepancy concerning travellers, in this case, Ontario’s policy regarding healthcare workers returning from abroad:

Some Ontario hospitals are still requiring staff to come to work immediately after travel, despite the province’s recommendation that everyone – including doctors and nurses – self-isolate after being abroad to minimize the spread of COVID-19 [68].

A reporter with the *Ottawa Citizen* identified an information discrepancy and concluded that “even public health officials are scrambling to keep up with the new messaging.” [69]. Travel and quarantine are just one set of confusing guidelines, which also involved issues such as how should ‘social bubbles’ be constituted, who should get tested and when, the allowable size of social gatherings, and so forth. Another source of confusion was the use of face masks, primarily due to the change in directives. Recommendations went from Dr. Tam not recommending their use for healthy individuals to recommending them as an extra measure of precaution, and then to local governments mandating their use in public indoor spaces. While the Public Health Agency of Canada revised its recommendation based on new evidence, the new guidelines were not always clearly communicated. In an editorial, for example, the *Globe and Mail* demanded better communication as facemask mandates varied significantly across jurisdictions:

I’m at the grocery store. Am I supposed to wear a mask? Many people are not. I’m on the bus. Am I supposed to wear a mask? Many people are. At some retailers, all employees are masked; in others, they aren’t. In some stores, customers must wear a mask; in others, none do. It’s time to replace a mass of vague and confusing suggestions with rules that are clear and simple [70].

As Canadians struggled to understand and follow ever-changing public health guidelines regarding travel, self-isolation, mask-wearing, and safe distancing, elected officials did not always follow their restrictions, causing anger and criticism. For example, despite asking Ontarians to limit contact to only people in their households, Premier Doug Ford spent Mother’s Day with his two adult daughters who do not reside in his household:

Reopening plans were also supposed to come with clear, easy-to-follow, science-based instructions on social guidelines. Instead, the Premier announced last week that domestic cleaners may now enter your home, but your grandma or sister still may not. The directive remained that Ontarians must stay two metres away from people outside of their households, even though Mr. Ford had two of his daughters – who live outside of his household – over at his home to celebrate Mother’s Day [56].

## Discussion

Uncertainty emerged in the news coverage of the COVID-19 pandemic in Canada as a “master frame” under which all the topics and guidelines fall. But underlying these debates was tension over exactly *how* scientific uncertainty was framed. In our analysis we found that reporters and commentators used four thematic faming strategies to challenge or support knowledge claims of uncertainty or decisions made under circumstances of evolving evidence. News coverage of emerging health threats affect public understanding of them [3, 24], and the four angles we identified in this study could deepen the feeling of uncertainty and confusion [24] about COVID-19 and reduce trust in guidance provided public health and elected officials. Specialist reporters sought to minimize uncertainty by focusing their coverage on peer-reviewed studies and the need for science-based policies, while non-specialist reporters aimed for balance in their stories, which resulted in pre-prints and other non-peer reviewed studies given the same validity as peer-reviewed ones. In trying to make sense of scientific contradictions and uncertainties, non-specialist reporters resorted to political frames that could deepen the feeling of uncertainty.

First, new scientific issues, such as novel health threats, carry considerable uncertainty, and journalists play a key role in making sense of the information and deciding whose voice will be heard [28, 71]. However, the contingent nature of the scientific evidence made it very challenging to determine what exactly the scientific knowledge around COVID-19 was, leading reporters to focus on new studies and emerging evidence. When faced with a novel scientific issue and being unable to assess the validity of scientific studies and information, reporters tend to cover a wide array of, sometimes contradicting, opinions in an effort to provide balanced and accurate coverage [28]. The copious number of studies being published on COVID-19, many of which were contradicting meant that new studies and emerging evidence did not provide reassurances, but in fact, underscored the general uncertainty regarding the novel coronavirus.

Second, reporters and commentators made sense of the scientific evidence and how it was used as a reason for action or inaction, by using the politicization of science frame. Reporters, particularly non-specialized ones, used a transparency and leadership frame through which changing public health guidelines was considered a political failure. Similarly, shifting guidelines and policies that contradicted some expert opinions were considered ‘typical’ political complacency and incompetence. Scientific uncertainty can serve political interests and justify both political action and inaction [16]. Some policy-makers appeal to uncertainty to justify lack of political action – for example, not mandating masks – while others may appeal to scientific uncertainty to invoke the need for precautionary principle [16]. While public health guidelines and the pandemic response were expected to be evidence-based, both specialist and non-specialist reporters, as well as commentators did not consider scientific uncertainty as enough reason to maintain the status quo. Instead, they condemned elected officials for not having applied a precautionary principle.

Third, uncertainty was exacerbated in the news coverage by putting the nature of the scientific process on display to the public and the use of the duelling experts frame. While health and science reporters explained the difference in validity between pre-prints and peer-reviewed studies, including reports of contradicting results, other reporters and commentators exacerbated the feeling of uncertainty by using the duelling experts frame. Scientific uncertainty led to reports of experts of equal stature openly disagreeing about which studies were valid and what ought to be done [16]. This framing then “reduces science to just another playground for competing ideologies” (16., p.40) and erodes trust in science. The abundance of scientific studies on COVID-19 published in the first months of the pandemic led some non-specialist journalists to write about pre-prints as if their findings were as valid as those of peer-reviewed studies. This lack of nuance in reporting scientific research can negatively impact public perception of the risk of COVID-19 and public health guidelines, thus pointing to the need for better science communication training for journalists.

Fourth, many reporters -both specialist and non-specialist- and columnists found the constant policy and regulation changes frustrating. This frustration was not due to the changes themselves but to poor communication for why these changes were necessary. Even health reporters, who devoted many words to explain how scientific knowledge advances by revising itself, expressed frustration over poor communication or confusing messages. Consequently, the uncertainty “master frame” governing the pandemic response was not solely presented as a scientific problem but also a political one.

### Implications for practice

The large scale of the pandemic and the abundance of news stories to be covered resulted in many non-specialist reporters having to write about health and science, and these reporters brought their analysis of politics, economy, sport, and other non-scientific specializations to bear on the uncertainties of COVID-19. However, political frames were also used by specialist reporters, who considered that the general sense of uncertainty was deepened by ineffective public health communication.

Health news play a key definitional role for health risks such as COVID-19. Studies have found that when journalists lack understanding about the scientific method and scientific uncertainty, science news stories tend to focus on political debate and simply report on opposing views and dramatization of the issue (c.f [72, 73].). To improve science reporting in an era in which specialization in news media is rare, Patterson [74] suggests the practice of knowledge-based journalism, i.e. training journalists to go beyond traditional reporting skills (e.g. interviewing, investigating, storytelling, etc.,) and also apply relevant specialized expertise when reporting on scientific issues. Knowledge-based journalism requires implementing science training programs for reporters, and there is evidence that such training help reporters feel more comfortable with scientific topics and more confident in their reporting abilities [75].

Based on our results, we advance three recommendations. First, during emergency situations, like the COVID-19 pandemic, media organizations should prioritise not contributing through their news coverage to additional uncertainty, panic, and controversy. Given the general uncertainty that comes with novel health threats, news organizations should aim to provide scientifically sound and consistent coverage and provide training for non-specialist journalists to ensure quality and consistency in their reporting during emergency situations. Such training should give non-specialist journalists the skills to evaluate the validity of scientific claims and avoid politicising evidence-based policy decisions. This could contribute to reducing confusion and controversy around science-based emergency response.

Second, public health officials and political leaders need to provide clear and consistent messages, as well as access to data and transparency, regarding infection prevention guidelines. Furthermore, public health officials should quickly engage in communication course corrections if original messages are missing the intended mark, and clearly explain the shift.

Finally, public health communicators should be aware of and more responsive to a variety of media reporters, who will bring different interpretative frames to their reporting. More care and effort are needed in these communication engagements to minimize inconsistencies, and account for the interpretive latitude that non-specialist reporters may bring forward in their stories.

One limitation of this study is that our classification of journalists as health/science reporters or non-specialist reporters was based on the reporters’ profiles in the newspaper websites and on information gathered from their individual websites. Our classification may, therefore, not accurately reflect the full extent of the reporters’ expertise. Another limitation of this study is that it focuses on news coverage of the first wave of the COVID-19 pandemic. A future examination could include news coverage of the second and third waves. Finally, this study focused on English language newspaper coverage in Canada. Further research could include also French language newspapers as well as other news outlets, or compare the Canadian news coverage of COVID-19 to news coverage of the pandemic in other countries.

## Conclusion

The COVID-19 pandemic brought the production of scientific knowledge onto the public agenda in real-time. News media and commentators analysed the successes and failures of the pandemic response in real-time, bringing the process of scientific inquiry, which is also fraught with uncertainty, onto the public agenda. We found that uncertainty emerged as a “master frame” in the news coverage of the pandemic, with reporters and commentators expressing frustration over changing public health guidelines, which evolved as new scientific evidence emerged. This showed that most reporters lack understanding of the scientific process, which led to the politicization of the pandemic response in their reporting. Our study highlights the importance of managing scientific uncertainty in evolving science-policy situations. Public health communicators should be aware of and more responsive to a variety of media reporters, who will bring different interpretative frames to their reporting. More care and effort are needed in these communication engagements to minimize inconsistencies, uncertainty, and politicization.

## Data Availability

The datasets used and/or analysed during the current study are available from the corresponding author on reasonable request.

## Footnotes

1 The *Winnipeg Free* Press, *Toronto Star,* and *The Globe and* Mail are independently owned. *The Montreal Gazette* and the *Ottawa Citizen* are owned by PostMedia and CanWest. *Vancouver Sun* belongs to CanWest, and the *National Post* to PostMedia.
